# Acute Progressive Visual Loss in a Case of Acute Myeloid Leukemia: Challenges in the Utility of Molecular Tests in Early Diagnose of Cytomegalovirus Retinitis

**DOI:** 10.1155/2018/2840707

**Published:** 2018-01-31

**Authors:** Ali Amanati, Nader Shakibazad, Bahman Pourabbas, Mohammad Hossein Nowroozzadeh, Soheila Zareifar, Omid Reza Zekavat

**Affiliations:** ^1^Professor Alborzi Clinical Microbiology Research Center, Shiraz University of Medical Sciences, Shiraz, Iran; ^2^Hematology Research Center, Shiraz University of Medical Sciences, Shiraz, Iran; ^3^Poostchi Eye Research Center, Shiraz University of Medical Sciences, Shiraz, Iran

## Abstract

Cytomegalovirus (CMV) retinitis is one of the rare but debilitating presentations of the CMV infection in children with leukemia. Herein, we report a 12-year-old boy with acute myeloid leukemia complicated by rapid progressive visual loss during relapse of leukemia. The definite diagnosis of CMV retinitis was made after vitreous aspiration. Despite prompt treatment and ophthalmologic intervention, he died because of AML relapse. Viral infections, especially cytomegalovirus infection, may present with vague clinical pictures during any time of chemotherapy, which may not be easily distinguishable from bacterial or fungal retinitis and also chemotherapy-induced retinopathies. Clinician should consider CMV retinitis in seropositive patients especially those without detectable viremia.

## 1. Introduction

Although the true incidence of CMV retinitis in non-human immunodeficiency virus (HIV) population such as leukemia is unknown [1, 2], but its occurrence is considered very rare especially in those with pediatric acute myeloid leukemia (AML) in the nontransplant setting [3]. The reported predisposing risk factors for CMV retinitis are older age, leukemia, systemic autoimmune disease, organ transplantation, lymphoma, diabetes mellitus, Good syndrome, immunosuppressive therapy, and multiple myeloma [3].

Sight-threatening emergencies may occur in any time during chemotherapy. Severe ophthalmic involvement, such as acute retinal necrosis (ARN) and progressive outer retinal necrosis (PORN), needs timely diagnose and prompt treatment. These are makeup of different etiologies, including infectious and noninfectious causes [4–7]. Chemotherapy-related ocular toxicity is one of the noninfectious etiologies of retinopathy in cancer patients [8, 9]. Certain antimicrobial agents such as voriconazole also may be accompanied with various degrees of ocular toxicity especially in whom without therapeutic drug monitoring (TDM) [10–12]. Posterior reversible encephalopathy syndrome (PRES) is also one of the important causes of acute visual loss during chemotherapy [13].

Prompt and regular ophthalmologic evaluation could guide empiric treatment, but in certain situations other diagnostic modalities help clinicians to make definite diagnosis. Herein, we report a case of relapsed AML on intensive chemotherapy, presented with acute visual loss, discuss the differential diagnosis, and describe the treatment choices.

## 2. Case Presentation

A 12-year-old boy a known case of relapsed AML has been admitted with impression of febrile neutropenia after the last course of chemotherapy on 16 April 2016. He received the FLAI regimen including fludarabine, cytarabine, and idarubicin (FLAI). After full sepsis workup, meropenem was started and cotrimoxazole (TMP/SMX) was continued with prophylaxis dosage (200 mg/12 hours by oral route). He received voriconazole as a fungal prophylaxis because of the previous history of proven pulmonary aspergillosis. Total white blood cell count was 200/mm^3^ (without countable neutrophils), hemoglobin was 10 g/dl, and platelet was 6000/mm^3^. First blood culture was positive for viridans streptococcus (by BACTEC system, time to detection: 9 hours) which was sensitive to vancomycin. Because high-grade fever continued after 48 hours of treatment and according to blood culture results, systemic vancomycin was added to antibacterial medications. The patient developed acute visual loss on 6th day of admission despite the appropriate type and dose of antibiotics. The neurological exam was normal. Retinal detachment and cancer associated retinopathy have been suggested as differential diagnoses in the first general ophthalmologist examination. The therapeutic dose of liposomal amphotericin B has been replaced with voriconazole.

Because high-grade fever was not resolved in 8th day of therapy (24 April), the blood sample was sent for a new culture, polymerase chain reaction (PCR; for aspergillosis, mucormycosis, candida, CMV, and tuberculosis), and enzyme-linked immunosorbent assay (ELIZA; for toxoplasmosis and cytomegalovirus antibody assay). A new chest X-ray has also been requested which revealed no new findings.

According to the results, toxoplasmosis IgM level was negative (0.1 IU/ml) and IgG level was at a borderline level (9.8 IU/ml). CMV blood PCR was reported to be 950  copy/mL. Anti-CMV antibody (IgG) was positive. Repeated blood PCR was negative for other fungal or viral pathogens.

Due to poor patient condition, spiral chest CT scan was requested on 10th day of admission with normal result. Rifampin has been added to the treatment regimen due to deteriorating patient's condition to achieve additive anti-Gram-positive synergistic effect. As the new blood culture results again revealed viridans streptococcus, which is susceptible to linezolid, linezolid has thus been replaced with vancomycin because of persistent bacteremia on 12th day of admission.

Lumbar puncture was performed on 29 April with total cells of 50/mm^3^ (white blood cells, 40/mm^3^ (polymorphonuclear leukocytes, 40%); red blood cells, 10/mm^3^), glucose of 50 mg/dl, protein of 48 mg/dl, and lactate dehydrogenase of 40 IU/L. Cytology report of cerebrospinal fluid (CSF) was positive for blasts, and PCR was negative for all viral and fungal suspicious agents.

**Figure 1:**
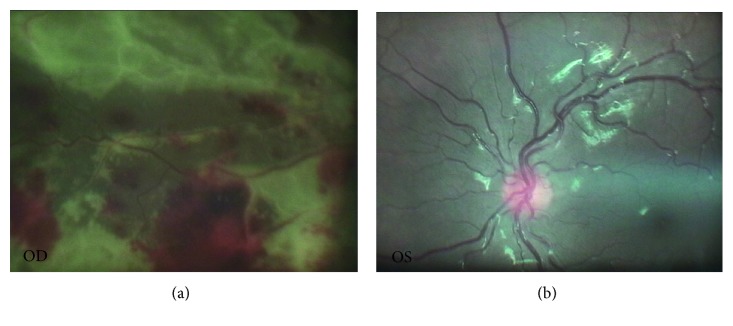
Diffuse retinal hemorrhage with patchy focal necrosis in the right eye and normal left eye.

Along with the patient's general condition, the patient complained of deteriorated visual function in the right eye. Therefore, a second ophthalmologist consult with a vitreoretinal specialist has been performed on 16th day of admission. The consultant ophthalmologist reported fluffy lesions with diffuse retinal hemorrhage and patchy focal necrosis (brush fire pattern) in the right eye and normal left eye (Figure 1). Thus, with first impression of cytomegalovirus (CMV) retinitis, vitreous aspiration was performed under anesthesia, and the sample was sent to the laboratory for PCR analysis. DNA was extracted from 100 *µ*L ocular fluid by using High Pure Viral Nucleic Acid Kit (Roche Diagnostic GmbH, Germany) according to the instruction manual. Cytomegalovirus viral load was determined using a genesig quantitative real-time PCR kit (Primer Design Ltd. TM, Advanced Kit, United Kingdom) according to the manufacturer's instruction. The target sequence (glycoprotein B) has previously been shown to be a good genetic marker for CMV in other real-time PCR-based studies [14]. This quantitative PCR assay was sensitive enough to detect 10 copies of CMV genomic DNA. Real-time PCR was performed using an ABI Step One Plus System (Applied Biosystems, Foster City, CA) with standard reagents (TaqMan Gene Expression Master Mix, Applied Biosystems, UK), which showed positive results for CMV (5000 copy/ml). Upon confirmation, the patient received 2 intravitreal injections of ganciclovir 1 week apart with adjunctive systemic antiviral therapy with ganciclovir 5 mg/kg twice daily on 24th day of admission for 2 weeks as induction. In addition, our plan was to continue for about 4 weeks.

On 34th day after admission, a new blood sample was requested for CMV PCR, which was shown 1150 copy/ml of CMV DNA in plasma without significant change in baseline CMV viral load.

Because of severe retinal necrosis and hole formation at some retinal areas, barrier argon laser photocoagulation was suggested and performed by the responsible ophthalmologist. The final blood culture became negative, but finally he was died due to refractoriness of leukemia and poor response to chemotherapy 8 weeks after admission.

## 3. Discussion

Chemotherapy-related retinopathy, retinal hemorrhage due to bleeding tendency, drug toxicity (voriconazole overdose), and infectious retinitis (viral, bacterial, or fungal) are among the most common causes of the sudden onset visual loss in a child with leukemia.

The most common side effect (30%) of oral and intravenous voriconazole administration is photopsia, or visual disturbances (patients describe increased brightness or blurred vision) that are transient and reversible. Moreover, color blindness and night blindness have been reported [15]. Rarely, sudden blindness is reported as a severe side effect of intravenous voriconazole therapy (but not oral formulation). It should be mentioned that our patient was received oral voriconazole [16].

Although CMV retinitis is less common than other etiologic causes of acute visual loss in acute leukemia [17–19], high level of suspicion, timely diagnosis, and prompt treatment could prevent permanent visual loss [19].

Currently, there is consensus regarding withholding ganciclovir in severely neutropenic patients, but ganciclovir still is the drug of choice in management of CMV infections including CMV retinitis [20]. It seems reasonable to treat the patient when CMV is the main etiologic cause of acute visual loss. Topical and systemic ganciclovir could save vision specially when considerable viremia is present (3- to 5-fold increase in the baseline CMV viral load or in case of CMV viral load of more than 2000 copy/ml) [21]. It should also be kept in mind that systemic CMV infection itself may exaggerate duration and depth of neutropenia by secondary bone marrow suppression [22, 23]. The risk of treatment with ganciclovir should be weighed against the benefits in such a situation. Although available data have been confirmed direct association between high CMV viral load and development of CMV retinitis in patients with CMV viremia after hematopoietic stem cell transplantation (HSCT) [24], there is lack of enough evidence to support this relationship in leukemia. Based on small case series, nearly all reported cases had viremia at the time of diagnosis [19]. Antiviral therapy usually is continued for 4–6 weeks.

As evident in our case, relying only on CMV viral load in the blood may postpone correct diagnose of CMV retinitis and delay timely antiviral treatment. Finally, it is necessary to emphasize that the diagnosis of CMV retinitis was made on the basis of ophthalmological findings suggesting typical diffuse retinal hemorrhage and patchy focal necrosis.

## 4. Conclusion

Although bacterial infections are the main cause of infectious complications in children with leukemia, leukemic patients are at increased risk of other opportunistic infections during the course of chemotherapy, such as invasive fungal and viral infections. Viral infections, especially cytomegalovirus, may present by vague clinical picture, which may not be distinguishable easily.

Clinician who involves in the management of patients with leukemia should be aware of these rare complications.
